# Disrupted Small-World Brain Networks in Moderate Alzheimer's Disease: A Resting-State fMRI Study

**DOI:** 10.1371/journal.pone.0033540

**Published:** 2012-03-23

**Authors:** Xiaohu Zhao, Yong Liu, Xiangbin Wang, Bing Liu, Qian Xi, Qihao Guo, Hong Jiang, Tianzi Jiang, Peijun Wang

**Affiliations:** 1 Imaging Department, TongJi University, TongJi Hospital Shanghai, China; 2 LIAMA Center for Computational Medicine, National Laboratory of Pattern Recognition, Institute of Automation, the Chinese Academy of Sciences, Beijing, China; 3 State Key Laboratory of Medical Neurobiology, Department of Neurology, Institute of Neurology, Huashan Hospital, Shanghai Medical College, Fudan University, Shanghai, China; 4 Key Laboratory for NeuroInformation of Ministry of Education, School of Life Science and Technology, University of Electronic Science and Technology of China, Chengdu, China; 5 The Queensland Brain Institute, The University of Queensland, Brisbane, Australia; Indiana University, United States of America

## Abstract

The small-world organization has been hypothesized to reflect a balance between local processing and global integration in the human brain. Previous multimodal imaging studies have consistently demonstrated that the topological architecture of the brain network is disrupted in Alzheimer's disease (AD). However, these studies have reported inconsistent results regarding the topological properties of brain alterations in AD. One potential explanation for these inconsistent results lies with the diverse homogeneity and distinct progressive stages of the AD involved in these studies, which are thought to be critical factors that might affect the results. We investigated the topological properties of brain functional networks derived from resting functional magnetic resonance imaging (fMRI) of carefully selected moderate AD patients and normal controls (NCs). Our results showed that the topological properties were found to be disrupted in AD patients, which showing increased local efficiency but decreased global efficiency. We found that the altered brain regions are mainly located in the default mode network, the temporal lobe and certain subcortical regions that are closely associated with the neuropathological changes in AD. Of note, our exploratory study revealed that the ApoE genotype modulates brain network properties, especially in AD patients.

## Introduction

Alzheimer's disease (AD) is the leading cause of intellectual impairment in the elderly worldwide [1], [2], [3]. In its early stages, the most commonly recognized symptom is an inability to acquire new memories, such as difficulty in recalling recently observed facts, which is commonly referred to as the loss of episodic memory. As the disease progresses, extensive cognitive impairments begin to manifest, including language breakdown and long-term memory loss. Eventually, most brain functions deteriorate, ultimately leading to death. The neural basis underlying the functional damage is not yet fully understood. Recent studies based on multimodal imaging have provided evidence supporting the notion of AD as a disconnection syndrome [4], [5], [6].

Information interactions between interconnected brain regions are believed to be a basis of human cognitive processes [7], [8]. Networks have been used to model the brain and provide a new tool for understanding functional integration and segregation in the human brain and also the pathogenesis and treatment of neurological disorders [8], [9], [10], [11]. Elucidation of the complexity of brain networks will offer fundamental new insights into the general organizational principles of neurological functions from both global and integrative perspectives [8], [12], [13], [14]. Characterizing the underlying architecture of brain networks is an important issue in neuroscience.

Previous brain network studies of AD patients have revealed that their cognitive functional deficits may be due to abnormalities in the connectivity between different brain areas, although there is no consensus as to what the alteration pattern is [15], [16], [17], [18], [19], [20], [21]. In 2007, using EEG data, Stam and colleagues found that although the network clustering coefficient was unchanged in AD patients, the patients displayed a longer characteristic path length [19]. However, in 2008, using fMRI data, Supekar and colleagues found that AD patients had a lower clustering coefficient and no change in characteristic path length [20]. In a structural imaging study the same year, He and colleagues found a higher clustering coefficient and longer characteristic path length in the brain structural networks of AD patients [17]. In 2009, using MEG data, Stam and colleagues found a lower clustering coefficient and higher characteristic path length in the brain network in AD patients [21]. Furthermore, in 2010, using resting-state fMRI data, Sanz-Arigita and colleagues found the clustering coefficient to be unchanged but a lower average shortest path in AD patients [18]. In the same year, using structural MRI data, Yao and colleagues found a higher clustering coefficient and longer average shortest path length in AD patients; the authors also found that network topological measures in mild cognitive impairment (MCI) patients were between those of AD and NC groups [15]. It should be noted that until now, studies of the altered brain network pattern in AD patients have not produced consistent results. With these studies, researchers do not obtain consistent results which may arise from the differences in the groups of subjects, different measurements and the image modalities used. As previously established, patients at different stages may manifest different behavioral symptoms with distinct underlying neural mechanisms [22], [23], [24]. Thus, a study focused on a group of subjects with a specific disease stage (for example, mild, moderate or severe AD) will help us understand the network alteration in AD. In addition, the apolipoprotein E (ApoE) gene, located on chromosome 19, is a major susceptibility gene and is most clearly linked to late-onset AD. ApoE4 is the risk allele of the ApoE gene in AD [25]. An increasing amount of evidence has indicated that ApoE4 modulates the brain activity of both normal aging and AD patients as measured by fMRI [26], [27], [28], [29]. However, to our knowledge, the question of whether the ApoE gene affects the topological properties of AD in the brain has not yet been studied.

In the current study, we specifically focused on moderate AD patients to directly investigate the hypothesis that the brain network of AD is characterized by the disruption of efficient small-world topological properties based on resting-state fMRI data. First, binary brain networks of individual brains were constructed with 90 brain regions as nodes extracted by an automated anatomical labeling (AAL) template [30] with inter-regional functional connectivity as edges. Second, the topological parameters of the brain network (clustering coefficient, shortest path length, global efficiency and local efficiency) were evaluated at different connection densities. Third, statistical differences between the AD and NC groups were evaluated at both global and nodal levels. Finally, to evaluate the effects of genotype on global network properties, we compared network properties between NCs without ApoE4 and ApoE4− and ApoE4+ AD patients.

## Materials and Methods

### Subjects

Only moderate AD patients and the age and education matched NC were included in the present study. All subjects gave voluntary and informed consents according to the standards set by the Ethics Committee of Tongji Hospital. The cognitive and neuropsychology tests were administered to each participant individually by a professional appraiser in the neuropsychological research center.

The cognitive abilities of the AD patients and controls were determined by Mini-Mental State Examination (MMSE) and Mattis Dementia Rating Scale(DRS) equivalent to that of the age-matched cognition level. The AD patients met the criteria for dementia as described by the National Institute of Neurological and Communicative Disorders and Stroke/Alzheimer Disease and Related Disorders Association (NINCDS-ADRDA) [31].The subjects were assessed clinically with the Clinical Dementia Rating (CDR) scale [32] and categorized as non-demented normal controls (NCs) (CDR = 0) and those with moderate stages of AD (CDR = 2). The inclusion criteria for both the normal controls and AD patients were as follows: 1) no anxiety or depressive disorders within a month; 2) normal vision and hearing; 3) cooperation with the cognitive tests; 4) age 50–85 years old with no constraints on education level; 5) no diagnosed stroke history; 6) no more than one lacunar infarction and no patchy or diffuse leukoaraiosis as determined by MRI examination.

The exclusion criteria included: 1) age under 50 years old or above 85 years old; 2) the presence of the following diseases or disease histories within a year: local brain injury, traumatic brain injury with loss of consciousness, confusion immediately following traumatic brain injury, serious mental diseases and alcohol or drug abuse; 3) obviously incomplete heart, liver, kidney or lung function; blood disorders; endocrine diseases; neurosyphilis; 4) clinical depression; 5) cancer; 6) excessive psychotropic drug use.

All subjects underwent a complete physical and neurological examination using an extensive battery of neuropsychological assessments and standard laboratory tests. Brain MRI scans of the AD patients showed no abnormalities other than brain atrophy.

### ApoE genotype

Venous blood samples from all subjects were added to EDTA anticoagulant after fMRI data acquisition. The technicians were blind to the diagnosis of the participants. ApoE genotypes were determined using standard methods [33].

The clinical and demographic data for the age- and gender-matched participants, excluding data from subjects with excessive head motion (see data preprocessing section), are shown in Table 1.

**Table 1:** Demographic, clinical and neuropsychological data.

	NC(n = 20)		AD(n = 33)			*P*
	ApoE−	ApoE+	ApoE−	ApoE+	*P* d	
Gender (M/F)	10/10		13/20			0.57^a^
	10/9	0/1	8/11	5/9	0.71	0.61^a^
Age (year)	63.0±5.8		66.2±9.6			0.18^b^
	63.3±5.8	58	66.9±9.4	65.3±10.1	0.64	0.42^c^
MMSE	27.8±1.3		15.3±2.9			<0.001^b^
	27.7±1.3	29	15.3±3.0	15.2±2.9	0.92	<0.001^c^
DRS	132.3±5.0		96.0±10.8			<0.001^b^
	132.0±4.9	138	94.6±9.9	97.8±12.1	0.41	<0.001^c^

a Chi-square was used for gender comparisons.

b Two samples two sides *t*-test was used for age and neuropsychological tests comparisons between AD and NC.

c One-way ANOVA was performed for age and neuropsychological tests comparisons.

d Two samples two sides *t*-test was used for age and neuropsychological tests comparisons between ApoE− and ApoE+ in AD group.

MMSE, Mini-Mental State Examination, DRS, Dementia Rating Scale.

Gray background means the detail statistical of the subject divided by using ApoE genotype.

Demographic, clinical and neuropsychological data in normal control patients (NCs) and Alzheimer's disease patients (AD).

### Data acquisition

Images were scanned on an American Marconi 1.5T EDGE ECLIPSE superconducting MRI system in the department of radiology of Tongji Hospital of Tongji University, Shanghai. Resting-state BOLD-fMRI was collected axially using an echo-planar imaging (EPI) sequence with the following parameters: repetition time (TR) = 2000 ms, echo time (TE) = 40 ms, flip angle (FA) = 90°, field of view (FOV) = 24 cm×24 cm, matrix = 64×64, NEX = 1, slices = 21, thickness = 6 mm, gap = 1 mm. The scan lasted for 320 seconds. The subjects were instructed to keep their eyes closed, relax their minds and remain as motionless as possible during the data acquisition. Rubber earplugs were used to reduce noise, and foam cushioning was used to fix the head to reduce motion artifacts.

### Data preprocessing

Unless specifically stated otherwise, all the preprocessing was carried out using statistical parametric mapping (SPM8, http://www.fil.ion.ucl.ac.uk/spm). The first 5 images were discarded in consideration of magnetization equilibrium. The remaining 155 images were corrected for the acquisition time delay among different slices, and then the images were realigned to the first volume for head-motion correction. The fMRI images were further spatially normalized to the Montreal Neurological Institute (MNI) EPI template and resampled to a 2-mm cubic voxel. Several sources of spurious variance including the estimated motion parameters, the linear drift, and the average time series in the cerebrospinal fluid and white matter regions were removed from the data through linear regression. Finally, temporal band-pass filtering (0.01≤f≤0.08 HZ) was performed to reduce the effects of low-frequency drift and high-frequency noise [34], [35].

The time course of head motion was obtained by estimating the translations in each direction and the rotations in angular motion about each axis for each of the 155 consecutive volumes. All the subjects included in this study exhibited a maximum displacement of less than 3 mm (smaller than the size of a voxel in plane) at each axis and an angular motion of less than 3° for each axis. Data from two subjects were excluded due to excessive motion. Furthermore, for exclusion the influence of head motion on functional connectivity results, an extra evaluation of the movement parameter between AD and NC group have been performed in line with the procedures described in Van Dijk et al. [36]

### Anatomical parcellation

The registered fMRI data were segmented into 90 regions (45 for each hemisphere, Table S1) using an automated anatomical labeling template [30], which has been used in several previous studies [20], [35], [37], [38], [39], [40]. For each subject, a representative time series of each individual region was then obtained by simply averaging the fMRI time series over all voxels in this region.

### Graph theoretical analysis

#### Brain network construction

The *Pearson* correlation coefficients of each area were calculated for each pair of 90 functionally connected regions. To simplify the statistical calculation, a Fisher r-to-z transformation was performed to increase the normality of the correlation matrix. Then, the absolute z values were converted into a binary connection matrix to make a graphic model of a brain network. That is, if the absolute z(i,j) (Fisher r-to-z of the partial correlation coefficient) of a pair of brain regions, i and j, exceeds a given threshold T, an edge is said to exist; otherwise it does not exist. Sparse networks of each subject were constructed using a minimum spanning tree method followed by global thresholds [41].

The degree of each node, D_i_, is defined as the number of nodes directly connected to the region i. The total number of edges in a graph, divided by the maximum possible number of edges, N(N-1)/2, that is, 
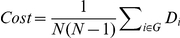
, is called the connection density or cost of the network, which measures how expensive it is to build the network [42]. The graphs were constructed over the whole range of connection densities or costs, from 4% to 40%, at 2% intervals. Global and nodal network properties were evaluated statistically over the range of 4–40%, and nodal properties were also analyzed at a connection density of 22%, at which the global efficiency showed the largest differences between AD and NC subjects.

#### Topological properties of the brain functional networks

All other topological properties considered were calculated using in-house software (Brat, www.ccm.org.cn/brat). These include the clustering coefficients (C_p_), shortest path length (L_p_), small-worldness, global efficiency (E_global_) and local efficiency (E_local_), each of which have been described previously and used in several prior studies [35], [38], [39]. Table 2 provides an overview of the parameters and their meaning in brain functional networks. A detailed description of these parameters can be found in the supplemental material (Text S1).

**Table 2:** Overview of the measurements and their meaning in brain functional network.

Character	Meaning
D_p_	degree of connectivity which evaluates the level of sparseness of a network
cost	cost of network
C_p_	clustering coefficient which measures the extent of a local cluster of the network
L_p_	path length which measures of the extent of average connectivity of the network
γ	γ = C_p_^real^/C_p_^rand^, the ratio of the clustering coefficients between real and random network
λ	λ = L_p_^real^/L_p_^rand^, the ratio of the path length between real and random network
σ	σ = γ/λ, scalar quantitative measurement of the small-wordness of a network
E_global_	a measure of the global efficiency of parallel information transfer in the network
E_local_	a measure of the fault tolerance of the network

Overview of brain functional network parameters and their meanings.

### Statistical analysis

Statistical comparisons of topological measures between the two groups were performed using a two-sample two-tailed *t*-test for each value over a wide range of connection densities (*P*<0.05). We determined the regional distribution of any statistically significant changes in the topological properties found between the two groups.

### Exploratory study of gene effect

To investigate the potential effect of ApoE genotype on network topological properties, we evaluated the differences between the NC ApoE4− group and the AD ApoE4− and ApoE4+ groups and those between the AD ApoE4− and ApoE4+ groups using a two-sample two-tailed *t*-test for each value over a wide range of connection densities (*P*<0.05). We determined the regional distribution of any statistically significant changes in the topological properties found between the two groups.

Caret v5.61 software was used to make cortical surface representations of the regional distributions of fixed group level network property alterations in the AD groups [43], [44]. The value plotted at a given point is the value of the template volume at a point below the surface at the level of the cortical layer.

## Results

### Cognitive and neuropsychology test

There were no significant differences in gender (*P* = 0.591) or age (*P* = 0.182) between the NC and AD groups. The MMSE scores of the NC group averaged 27.8±1.3, which fits the normal age-matched standard. The MMSE scores of the AD group averaged 15.3±2.9, which is significantly different from that of the NC group (*P*<0.001). The Mattis Dementia Rating Scale (DRS) scores of the NC group averaged 132.32±5.0 and those of the AD group averaged 96.00±10.8, and the scores also markedly differed from each other (*P*<0.001) (Table 1). There was one ApoE4+ NC subject, 19 ApoE4− NC subjects, 19 AD ApoE4− subjects and 14 AD ApoE4+ subjects. There were no significant differences in either gender or age between the NC ApoE4−, AD ApoE4− and AD ApoE4+ groups (Table 1). In addition, there were no significant differences in MMSE or DRS scores between the ApoE4− and ApoE4+ AD groups (Table 1).

### Evaluation of the movement parameter between AD and NC groups

The displacements of the MAD and NC groups are 0.89±0.39 mm and 0.80±0.60 degree, respectively, and the rotations of the MAD and NC groups are 0.69±0.33 mm and 0.52±0.30 degree, respectively. No significant differences were found between the experimental groups (two-sample, two-tailed *t*-test; *P* = 0.51 for displacement and *P* = 0.07 for rotation).

### Direct comparisons between the AD and NC groups

The mean functional connectivity matrix of each group was calculated by averaging the N×N (N = 90 in the present study) absolute connection matrix of all the subjects within the group. In the normal group, most of the strong functional connectivities (large z-scores) were between inter-hemispheric homogeneous regions, within a lobe, and between anatomically adjacent brain areas (Fig. S1). This functional connectivity pattern is consistent with many previous studies of whole brain functional connectivity in the resting-state [35], [38], [39], [45]. The AD group showed a similar functional connectivity pattern to that of the healthy group; however, the correlation strength was significantly altered (F_1, 51_ = 22.2, P<0.001).

### Altered topological properties of functional networks in AD subjects

**Figure 1:**
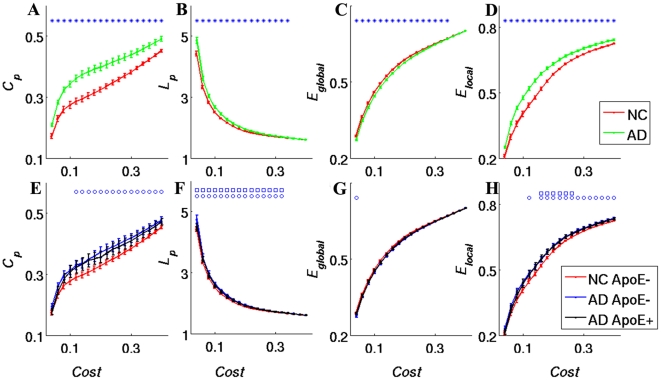
Change of network parameters as a function of connection density (Cost). Clustering coefficient (**A**), shortest path length (**B**), global efficiency (**C**) and local efficiency (**D**) of the AD (green line) and NC (red line) groups as a function of Cost. Clustering coefficient (**E**), shortest path length (**F**), global efficiency (**G**) and local efficiency (**H**) of the AD ApoE4+ (black line), AD ApoE4− (blue line) and NC ApoE4− (red line) groups as a function of Cost. The error bars correspond to the standard error of the mean. Blue asterisks indicate where the difference between the NC and AD groups is significant (*P*<0.05). Blue circles indicate points where the difference between the AD ApoE4− group and the NC ApoE4− group is significant (*P*<0.05). Blue squares indicate points where the difference between the AD ApoE4+ group and the NC ApoE4− group is significant (*P*<0.05). The difference between the AD ApoE− group and the AD ApoE+ group is not significant at any Cost (*P*>0.05).

In the range of 0.04≤Cost≤0.4, clustering coefficients (C_p_), shortest path length (L_p_), small-worldness, global efficiency (E_global_) and local efficiency (E_local_) values for the AD and NC groups were calculated and then compared using a two sample two-tailed *t*-test. With increasing connection density, C_p_, E_global_ and E_local_ all increased, whereas L_p_ decreased in both the AD and NC groups (Fig. 1). Across the entire threshold range, C_p_ and E_local_ values were notably higher in the AD group than in the NC group (*P*<0.05) (Fig. 1 A and D). In a wide threshold range (0.04∼0.34), the L_p_ of the AD group was also significantly greater than in the normal controls (*P*<0.05) (Fig. 1 B), but the E_global_ of the AD group was significantly lower than that of the NC group in this range (*P*<0.05) (Fig. 1C).

**Figure 2:**
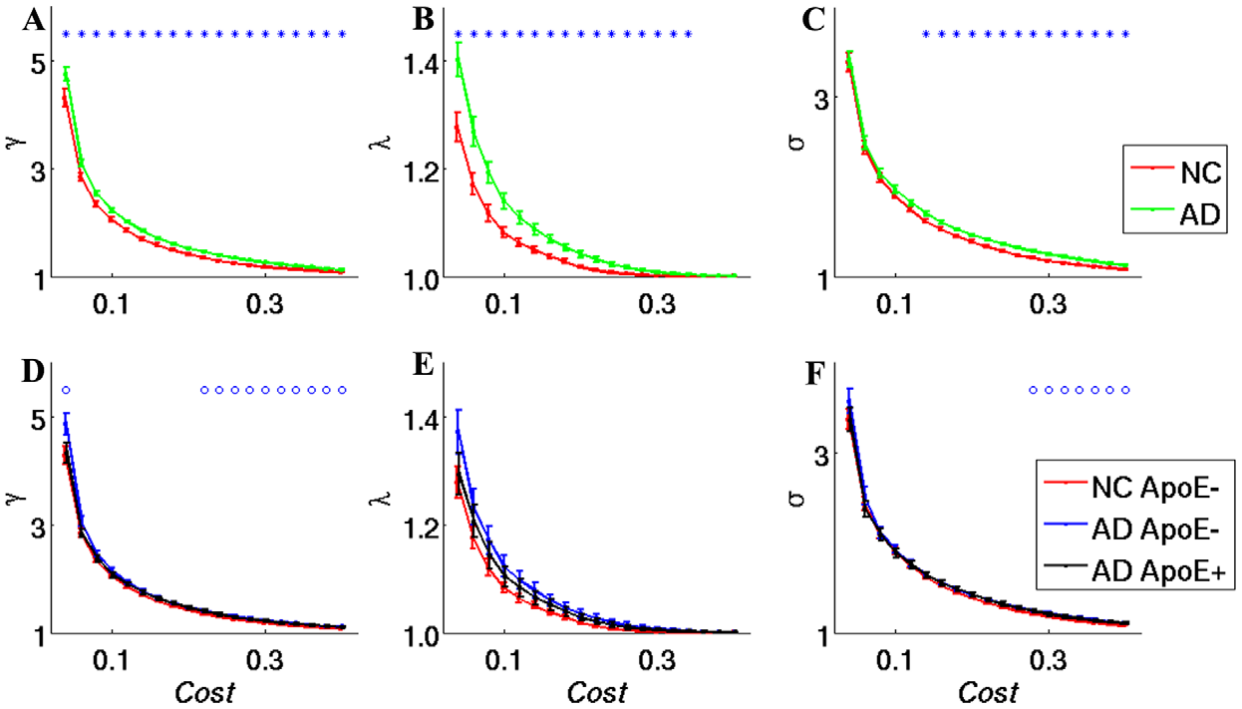
Change of small-world network definition parameters as a function of connection density (Cost).γ (**A**)**, λ** (**B**)**, and σ** (**C**) **of the AD (green line) and NC (red line) groups as a function of Cost.** γ (**D**), λ (**E**), and σ (**F**) of the AD ApoE4+ (black line), AD ApoE4− (blue line) and NC ApoE4− (red line) groups as a function of Cost. The error bars correspond to the standard error of the mean. Blue asterisks indicate points where the difference between the two groups is significant (*P*<0.05). Blue circles indicate points where the difference between the AD ApoE4− group and the NC ApoE4− groups is significant (*P*<0.05). No significant differences were found between the NC ApoE4− and AD ApoE4+ groups at any threshold (*P*>0.05) or between the ApoE4− and AD ApoE4+ groups.

The γ, λ and σ (a detailed definition can be found in Table 2 and the supplemental material) values of the brain network as a function of connection density within both groups is shown in Fig. 2. Both groups fit γ = C_p_^real^/C_p_^rand^>1 and λ = L_p_^real^/L_p_^rand^≈1 (Fig. 2 A and B). Thus, the functional networks of AD patients and NCs fit the definition of small-worldness [46]. The γ and λ values were significantly higher in the AD group over nearly the entire range of connection density (Fig. 2 A and B). The σ values of the AD group were significantly larger than that of the NC group in the range of 0.14≤Cost≤0.4(Fig. 2 C).

### Distribution of brain regions with altered network properties in AD subjects

Two-sample two-tailed *t*-tests for each of the 90 regions was performed to further localize the nodes that demonstrated significant differences between the AD and normal control groups.

**Figure 3:**
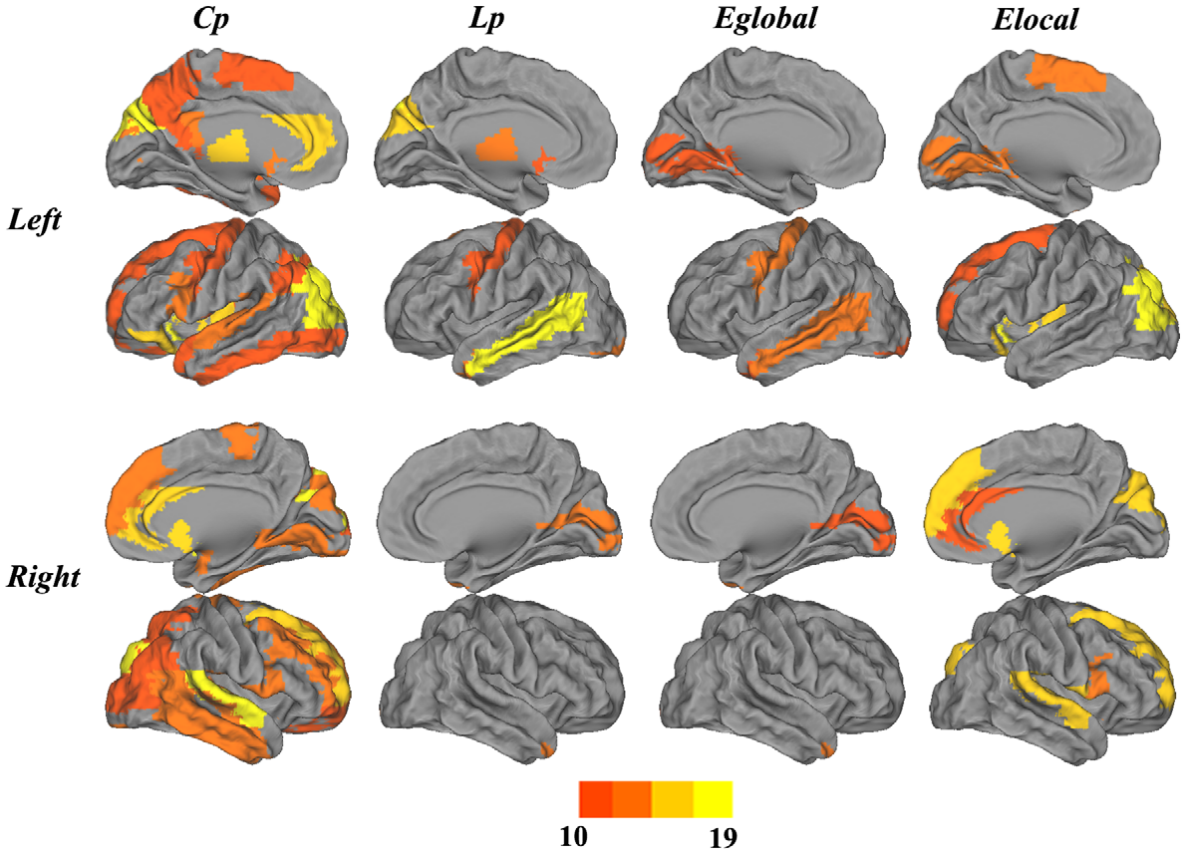
Surface rendering of the distribution of altered nodes (*P*<0.05). Nodes with altered times greater than 10 across the 19 comparisons are shown. Colored bars indicate altered times. Regions showing significant alterations in C_p_ and E_local_ are widely distributed across the brain, especially in the default mode network (such as the PCC_R, ACC, IFGoper, and SFG), regions in the temporal lobe (such as the STG) and regions in the subcortical structure (such as the THA_R, PAL_L and PUT_R). Significant alterations in E_global_ and L_p_ are primarily found in temporal lobe regions, such as the MTGp and MTG_R, and sensory motor regions, such as the SMA_R and PreCG_R.

Fig. 3 shows the frequencies of the altered nodes that were found across the 19 threshold times (*P*<0.05) in the AD patients (Fig. S2). As shown in Fig. 3, the regions showing significant alterations in C_p_ and E_local_ are widely distributed across the brain, especially in the default mode network, which is composed of the right posterior cingulate gyrus (PCC_R), the anterior cingulate and paracingulate gyrus (ACC), the opercular part of the inferior frontal gyrus (IFGoper), the superior frontal gyrus (SFG), regions in the temporal lobe such as the superior temporal gyrus (STG), and regions in the subcortical structure such as the right thalamus (THA_R), left lenticular nucleus pallidum (PAL_L) and right lenticular nucleus putamen (PUT_R). The regions showing significant alterations in E_global_ and L_p_ are distributed primarily in regions of the temporal lobe, such as the middle temporal gyrus temporal pole (MTGp) and right middle temporal gyrus (MTG_R), and sensory motor regions, such as the right supplementary motor area (SMA_R) and right precentral gyrus (PreCG_R).

**Figure 4:**
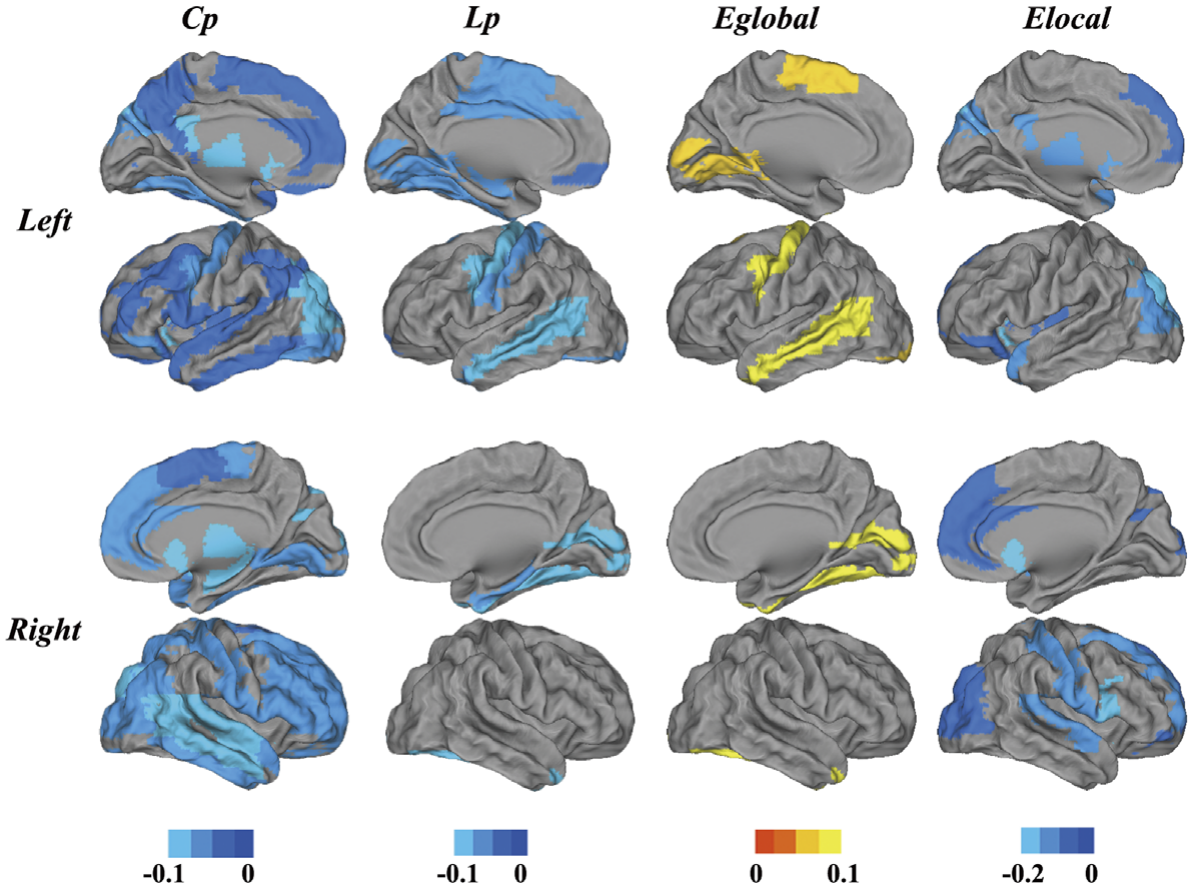
Surface rendering of the distribution of altered nodes at a connection density of 22%. **Colored bars indicate differences in network properties between the NC and AD groups.** Blue indicates regions showing an increase in the AD group but not the NCs. Yellow indicates regions showing a decrease in the AD group but not the NCs. In the AD group, the regions showing significant increases in C_p_, L_p_ and E_local_ are widely distributed across the brain, especially in default mode network regions such as the ACC, PCC, MPFC, HIP and IPL; regions in the temporal lobe such as the STGp/MTGp; and regions in the subcortical structure such as the THA, INS and PUT. The regions showing significant E_global_ decreases in AD are distributed primarily in the bilateral MTG and motor areas such as the SMA_R and PreCG_R.

We further explored the brain regions where the topological parameters were significantly different between AD patients and NCs at the cost of 0.22 (global efficiency showed the most prominent differences at this connection density). As shown in Fig. 4 (A/B/D), most of network topological properties (C_p_, L_p_ and E_local_) were found to be increased in AD patients relative to controls across widely distributed regions (also see Fig. S3). We generally grouped the altered regions into three clusters. The first cluster is the default mode network (Fig. 4); nearly all the regions belonging to a typical default mode network can be identified in our results, such as the ACC, PCC, middle prefrontal cortex (MPFC), hippocampus (HIP) and inferior parietal cortex (IPL). The second cluster includes parts of the subcortical structure such as the thalamus (THA), lenticular nucleus putamen (PUT) and INS (Fig. 4). The third cluster consists of regions of the temporal lobe such as the superior/middle temporal gyrus temporal pole (STGp/MTGp) and the bilateral middle temporal gyrus (MTG), which showed significantly decreased E_global_ in AD patients (Fig. 4C). E_global_ was also found to be decreased in AD patients in motor areas such as SMA_R and PreCG_R.

### The ApoE4 gene modulates global network properties

**Figure 5:**
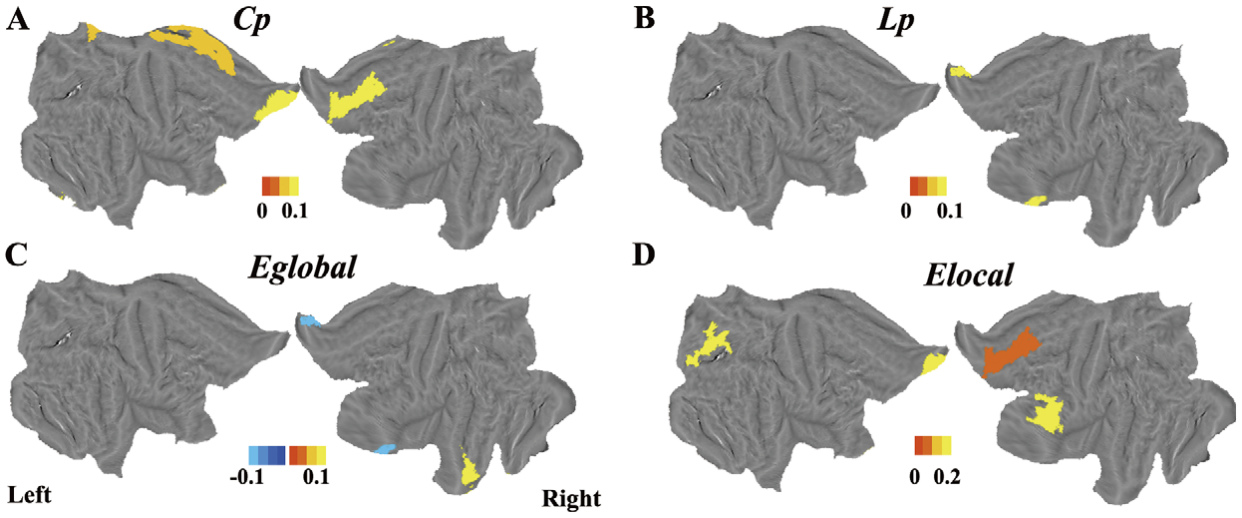
Surface rendering of the distribution of nodes differing between the AD ApoE4+ and ApoE4− groups. Colored bars indicate differences in network properties between the two groups. Blue indicates regions showing an increase in the AD ApoE4+ group. Yellow indicates regions showing a decrease in the AD ApoE4+ group. The ApoE4− group displayed higher C_p_, higher E_local_, and longer L_p_ in the temporal, frontal and medial prefrontal cortexes, and lower E_global_ in the frontal cortex.

Compared with the NC ApoE4− subjects, the AD patients had significantly higher C_p_, higher E_local_, and longer L_p_, but lower E_global_ (Fig. 1, E–H) regardless of their ApoE4 status (*P*<0.05). Relative to the AD ApoE4− group, the AD ApoE4+ group showed lower C_p_, lower E_local_, shorter L_p_, and higher E_global_. Although these differences did not reach statistical significance, the trend was clear (Fig. 1, E, F, G, H, Fig. 2, D, E, F). In addition, the brain regions where the topological parameters significantly differ between the patients carrying and not carrying ApoE4 gene were investigated. The AD ApoE4− patients displayed higher C_p_, higher E_local_, and longer L_p_ in the temporal cortex, frontal cortex and medial prefrontal cortex, and they displayed lower E_global_ in the frontal cortex (Fig. 5 D, E, F, Fig. S4).

## Discussion

Our results revealed that the topological properties of the brain networks in the moderate AD group were disrupted compared to those in the normal controls. Specifically, the altered regions were mainly distributed in the default mode network, the temporal lobe and subcortical structures. More importantly, we also evaluated the effects of genotype on network properties, especially in the AD group.

### Small-world topology in normal individuals

The human brain is a functionally specialized organ with anatomically distinct structures. Cognition requires a high level of functional interaction between brain regions to support daily activities. Recent studies with noninvasive brain imaging technologies such as MRI, EEG, and MEG have demonstrated that the human brain's structural and functional networks have small-world properties [9], [10], [47]. The brain networks in NCs and AD patients fit the features of small-world networks, suggesting that the human brain supports rapid real-time integration of information across segregated sensory brain regions [12], [13] to confer resilience against pathological attacks [39] and to maximize efficiency at a minimal cost for effective information processing between different brain regions [38], irrespective of age or illness status.

### Altered small-world topology in AD patients

Our investigation revealed that network topological properties are altered in AD patients, consistent with previous studies [15], [17], [18], [19], [20], [21], [48].

C_p_ is a measure of local network connectivity. It reflects the local efficiency and error tolerance [49] of a network. Higher network clustering coefficients indicate more concentrated clustering of local connections and stronger local information processing capacity [10]. The C_p_ of brain functional networks was found to be higher in AD patients, indicating that these patients have stronger local information processing capacity. This finding is consistent with previous structural network studies in AD [15], [17].

The average shortest path length (L_p_) of a network reflects how the network connects internally [46]. In brain networks, the shortest path ensures the effective integration and fast transmission of information between distant brain areas. Generally speaking, the integration and transmission of information are the bases of cognitive processing [8], [11], [13]. Our results demonstrate that the average shortest path of the brain functional networks in AD patients was significantly greater than that in NCs, indicating that the long distance information integration and transmission capacity of neurons is reduced in AD patients [15], [18], [19]. Together with the lower global efficiency in AD, these results suggest that information transfer between brain regions is more difficult in AD patients. Our results are consistent with several studies reporting attenuated long-distance functional connections and increased local functional connections in AD patients [50], [51], [52].

### Altered network properties at the node level in AD subjects

Comparisons of C_p_, L_p_, E_local_ and E_global_ provided us a perspective with which to investigate differences at the global level, and further nodal level comparisons localized the regions with significantly divergent brain network topological properties. We first explored the brain regions in which AD-related alterations were most likely to occur, and we further investigated the brain regions where the topological properties of AD patients differed significantly from those of the NCs. Our results revealed nearly overlapping patterns of brain regions (Fig. 3 and Fig. 4) where higher frequency alterations occurred, thereby showing significant differences between the two groups. We generally grouped these regions into three clusters of the default mode network, the subcortical structure and the temporal lobe. These regions had a higher probability of significantly abnormal topological properties in AD patients compared to NCs.

The default mode network has received growing attention over the last decade in neuroimaging studies [53], [54] investigating the functions of remembering the past, envisioning future events, and considering the thoughts and perspectives of other people [55], [56], [57], [58]. The relationships between the default mode network and AD and MCI have been extensively investigated using multiple imaging approaches including PET, structural MRI and fMRI [3], [53], [59]. In all instances, from metabolism changes to structural atrophy to functional abnormalities, these multiple modality methods have consistently revealed abnormal changes in the default mode network of AD patients. For example, previous studies using PET and single photon emission computed tomography techniques found that AD patients had abnormally low cerebral blood flow and low cerebral metabolic rates for glucose in many brain regions, including the parietal, temporal, and prefrontal cortices and the PCC [60], [61], [62], [63], [64]. Metabolic studies have revealed that the pattern of hypometabolism bears a striking resemblance to the regions comprising the default network [65], [66]. Structural MRI has shown gray matter loss in some regions belonging to the default network [67], [68]. Several fMRI studies [59], [65], [69], [70] have revealed consistent disruption with metabolic and structural changes in the default mode network in AD patients. Thus, our results complement and extend previous topological studies revealing a disrupted default network in AD patients.

Another important cluster in which the network properties are altered is the subcortical structure, particularly the thalamus and putamen. The thalamus is an important region with complex functions. In particular, every sensory system (with the exception of the olfactory system) includes a thalamic nucleus that receives sensory signals and sends them to the associated primary cortical region. The anterior and dorsal medial nuclei of the thalamus [71] and mammillo-thalamic tract [72] were found to be involved in episodic memory, which is specifically impaired in AD [73]. Indeed, a clinical case study and EEG studies have consistently demonstrated the important role of the human thalamus in language, executive functioning, attention and memory functions [74], [75]. Language, executive function, attention and memory functions have been shown to be broadly deteriorated in AD patients. Zarei and colleagues showed regional thalamic degeneration in AD patients by combining shape and structure connectivity analysis [76]. Wang and colleagues found that the functional connectivity pattern between the thalamus and the default mode network was significantly altered in early stages of AD [77]. The putamen has been found to be involved in reinforcement learning and implicit learning [78]. Alterations in the network properties of this subcortical structure are thought to be related to the episodic memory impairment in AD.

The temporal lobe is one of the largest lobes in the human brain with functions ranging from primary auditory sensation to high cognitive roles such as language, social cognition and memory [79]. The medial temporal lobe may be an early and profoundly involved area of neurofibrillary tangles, which is why a considerable amount of studies of the anatomic basis for memory impairment in AD has focused on the hippocampus and other medial temporal lobe structures [80]. Increasing evidence has shown that the superior temporal gyrus is equally important to social cognition, regulation of behavior, and the neural mechanisms of imitation [79]. Numerous studies have reliably revealed significant atrophy of the temporal gyrus in MCI and AD subjects relative to NCs [81]; these structural changes might reflect the pathological alterations in AD and thus may be related to the dysfunction observed in these patients. Of importance, most regions of the temporal lobe are involved in the default mode network, such as the hippocampus, lateral temporal cortex and the pole of the temporal cortex, which are attributed to the dorsal medial temporal lobe subsystem of the default mode [82]. As discussed above, the topological changes in the temporal lobe might be indirectly associated with the broad cognitive functional losses in AD such as the language impairment and (especially) the episodic memory impairment.

### ApoE genotype modulates topological properties in AD

It is well known that ApoE4 is a major risk factor for late-onset AD [83]. Previous fMRI studies have shown that the ApoE gene affects brain activity during different task in cognitively normal subjects [84], [85], [86] and in AD subjects [2], [87]. However, until now, it has remained unclear whether the ApoE4 gene affects the topological properties of the brain. Thus, in the current study, we aimed to investigate whether ApoE genotype modulates topological properties in AD patients. Here, we selected subjects who do not carry the ApoE4 allele as NCs because the frequency of ApoE4+ is lower in China [88] and Shanghai [33]. Our results showed that AD ApoE4+ patients had lower C_p_, lower E_local_, shorter L_p_ and higher E_global_ compared to ApoE4− patients. Although these differences did not reach statistical significance, the trend was clear, suggesting that significance could be achieved with a larger sample size. Our results are consistent with several MRI and behavioral studies in which AD ApoE4+ and ApoE4− patients showed different disruption patterns [26], . Thus, the differences in topological parameters between the two AD groups may reflect different neurological functional impairments. However, the underlying mechanism of such differences remains to be further explored.

### Methodological issues and further discussion

In the current study, we found that clustering coefficients, the shortest path length, local efficiency, and connection density were all elevated in AD patients, whereas global efficiency was lower. These findings are consistent with previous structural network studies [15], [17]. Our current results showed that the clustering coefficient and average shortest path length were both higher in AD patients than in NCs which might not completely align with two previous fMRI studies [18], [20]. Supekar and colleagues reported a lower clustering coefficient with no significant alteration of the average shortest path in AD patients [20]. In contrast, Sanz-Arigita and colleagues showed an unchanged clustering coefficient but a shorter average shortest path in AD patients [18]. Some confounding factors may contribute to this issue. One possibility is methodological differences in the data analyses (for examples, whether spatial smoothing was performed). The effects of local data smoothing can introduce differences between both groups examined due to the higher degree of cortical atrophy characteristic in AD [18], [20]. Another explanation is that there may be methodological differences in the functional connectivity measurements [Pearson's correlation (frequency 0.01–0.08 Hz) in our study, wavelet correlation (frequency 0.01–0.05 Hz) in Sepekar et al., and synchronization likelihood in Sanz-Arigita et al.]. Another important possible reason is the different subjects involved in these studies, especially with respect to the different progressive stages of AD. From a clinical perspective, the heterogeneity of AD and its distinct progressive stages are critical factors that can affect the results of AD studies. Patients at different stages may manifest different behavioral symptoms with different underlying neural mechanisms [22], [23], [24].

The present work mainly focused on moderate AD. Our first consideration is that the existing literature regarding the brain network analysis of AD patients did not clearly point out the progressive stages of AD patients (for example, early, moderate or severe). As discussed above, different AD stages may have different neural mechanisms. The AD patients at the moderate stage may present the typical brain changes and symptom manifested in AD, but with a slight cognitive impairment relative to the severe AD patients. The slight cognitive impairment allows the patients to cooperate in our fMRI study, such as better understanding and carrying out the experimental instructions. The topological alteration in this group will provide extensive additional evidence for the brain alterations present in AD.

We admit that we used the pure network theory to evaluate the altered network pattern in moderate AD subjects. In the present study, we only used the information of the strength of the functional connectivity to generate the binary network of each subject and then evaluate the network properties using graph theory. Such measurement will induce some information loss, such as the weight of functional connectivities, the distance between the two connected brain regions, etc. Thus, the local brain network information (such as local efficiency) here does not reflect the local brain area connections found in prior studies [41], [89], [90]. As previously established, the connection strength reflects the activity coherence, whereas the connection distance reflects the physical distance between regions. As anticipated from the results of prior studies [91], [92], the strength of functional connectivity between regions generally decreased as a non-linear function of increasing anatomical distance between regions. Further work will be needed to estimate the cortical distance information with greater precision, such as including information about white matter tract length or the physical distance between the functionally connected brain regions.

We did not perform a strict correction for multiple comparisons (such as a false discovery ratio); thus, we should be very careful when explaining our findings. To evaluate the robustness of our results, we have performed permutation tests. For each connection density, we have performed permutation statistical analysis for 10,000 trials. The permutation results were similar to our direct comparisons using the two-sample, two-tailed *t*-test (Table 3, 4). These additional findings suggest that our results are robust and credible.

**Table 3:** Statistical tests on the topological properties of the networks for all the groups after the permutation test.

Connect	Cluster coefficient		Path length		Global Efficiency		Local Efficiency	
Density	D_P	P_P	D_P	P_P	D_P	P_P	D_P	P_P
**0.040**	. 000030	0.00017	0.0027	0.0030	0.0024	0.0028	0.0001	0.0001660
**0.060**	0.00010	0.00002	0.0107	0.0096	0.0082	0.0066	0.0001	0.0000161
**0.080**	0.00010	0.00001	0.0121	0.0119	0.0103	0.0085	0.0002	0.0000035
**0.100**	0.00020	0.00005	0.0078	0.0072	0.0053	0.0048	0.0001	0.0000064
**0.120**	0.00010	0.00003	0.0074	0.0095	0.0049	0.0054	0.0001	0.0000016
**0.140**	0.00010	0.00002	0.0110	0.0123	0.0057	0.0065	0.0001	0.0000001
**0.160**	0.00010	0.00003	0.0043	0.0067	0.0040	0.0040	0.0001	0.0000001
**0.180**	0.00020	0.00004	0.0065	0.0069	0.0032	0.0042	0.0001	0.0000001
**0.200**	0.00020	0.00008	0.0028	0.0039	0.0020	0.0028	0.0001	0.0000015
**0.220**	0.00010	0.00010	0.0033	0.0049	0.0030	0.0034	0.0001	0.0000009
**0.240**	0.00020	0.00016	0.0043	0.0060	0.0038	0.0050	0.0001	0.0000048
**0.260**	0.00020	0.00016	0.0075	0.0099	0.0041	0.0084	0.0001	0.0000116
**0.280**	0.00020	0.00026	0.0089	0.0126	0.0084	0.0116	0.0001	0.0000605
**0.300**	0.00020	0.00024	0.0099	0.0180	0.0113	0.0167	0.0001	0.0000467
**0.320**	0.00050	0.00030	0.0157	0.0295	0.0176	0.0277	0.0001	0.0001362
**0.340**	0.00040	0.00038	0.0311	0.0468	0.0279	0.0450	0.0002	0.0002133
**0.360**	0.00040	0.00035	0.0508	0.0852	0.0464	0.0818	0.0003	0.0002098
**0.380**	0.00040	0.00050	0.0589	0.0943	0.0577	0.0936	0.0004	0.0003354
**0.400**	0.00050	0.00059	0.0612	0.0938	0.0571	0.0938	0.0002	0.0004387

D_P, the P value of the difference between the normal control and AD group.

P_P, the permutation P value of the difference between the normal control and AD group.

Statistical tests on the topological properties of the networks for all the groups of the permutation test.

**Table 4:** Statistical tests on the small-world properties of the networks for all the groups after the permutation test.

Connect	Gamma		Lambda		Sigma	
Density	D_P	P_P	D_P	P_P	D_P	P_P
**0.040**	0.03620	0.03604	0.00720	0.00756	0.84222	0.83874
**0.060**	0.01290	0.01393	0.01240	0.01295	0.70183	0.70459
**0.080**	0.00280	0.00406	0.01170	0.01254	0.55544	0.55638
**0.100**	0.00140	0.00171	0.00580	0.00669	0.29717	0.29083
**0.120**	0.00020	0.00015	0.00660	0.00758	0.08909	0.08932
**0.140**	0.00020	0.00007	0.00830	0.01045	0.02630	0.02455
**0.160**	0.00040	0.00015	0.00510	0.00542	0.01240	0.01264
**0.180**	0.00010	0.00004	0.00400	0.00484	0.00140	0.00179
**0.200**	0.00010	0.00007	0.00180	0.00270	0.00140	0.00128
**0.220**	0.00010	0.00001	0.00160	0.00335	0.00030	0.00016
**0.240**	0.00010	0.00002	0.00240	0.00441	0.00010	0.00007
**0.260**	0.00020	0.00001	0.00550	0.00837	0.00010	0.00002
**0.280**	0.00010	0.00003	0.00730	0.01058	0.00010	0.00003
**0.300**	0.00010	0.00003	0.00730	0.01298	0.00020	0.00003
**0.320**	0.00010	0.00002	0.01580	0.02241	0.00010	0.00002
**0.340**	0.00010	0.00004	0.02300	0.03664	0.00010	0.00004
**0.360**	0.00010	0.00006	0.03470	0.05928	0.00020	0.00006
**0.380**	0.00010	0.00005	0.03830	0.05572	0.00010	0.00005
**0.400**	0.00030	0.00013	0.04180	0.06449	0.00020	0.00012

D_P, the P value of the difference between the normal control and AD group.

P_P, the permutation P value of the difference between the normal control and AD group.

Statistical tests on the small-world properties of the networks for all the groups of the permutation test.

### Conclusions

In summary, we have investigated the topological properties of human brain functional networks in moderate AD patients using resting-state fMRI. Brain functional networks have efficient small-world properties that support efficient parallel information transfer at a relatively low cost in normal controls. However, this functional organizational mechanism is disturbed in patients with moderate AD, especially in patients with the ApoE4− genotype. This finding is consistent with the hypothesis of dysfunctional integration in the brains of AD patients.

## Supporting Information

Figure S1 **Mean z-score matrices for normal and AD group.** Each figure shows a 90×90 square matrix, where the x and y axes correspond to the regions listed in Table S1, and where each entry indicates the mean strength of the functional connectivity between each pair of brain regions. The diagonal running from the upper left to the lower right is intentionally set to zero. The z-score of the functional connectivity is indicated with a colored bar.
(TIF)

Figure S2 **The frequencies distributions of altered brain areas in AD group of the 19 different thresholds.** Y axes presents significantly altered brain areas. The traverse axes correspond to the frequency of significant differences happened between the two groups from 0.04–0.40 at 0.02 intervals. Clustering coefficient (blue), local efficiency (green), shortest path length (yellow), and global efficiency(brown).
(TIF)

Figure S3 **The altered brain areas between AD group (red) and NC group(blue) at the cost of 22%. A, clustering coefficient; B, shortest path length; C, global efficiency; D, local efficiency.**
(TIF)

Figure S4 **Brain areas showed significant alteration in network properties between ApoE+(red) and ApoE−(blue) groups in AD at the cost of 22%.** A, clustering coefficient; B, shortest path length; C, global efficiency; D, local efficiency.
(TIF)

Table S1 **Cortical and subcortical regions defined in Automated Anatomical Labeling template in standard stereotaxic space.**
(DOCX)
