# Supplementary figures and images for: Disrupted Small-World Brain Networks in Moderate Alzheimer's Disease: A Resting-State fMRI Study
Source: PLoS One. 2012 Mar 23;7(3):e33540. doi: 10.1371/journal.pone.0033540 (PMC3311642; doi:10.1371/journal.pone.0033540)

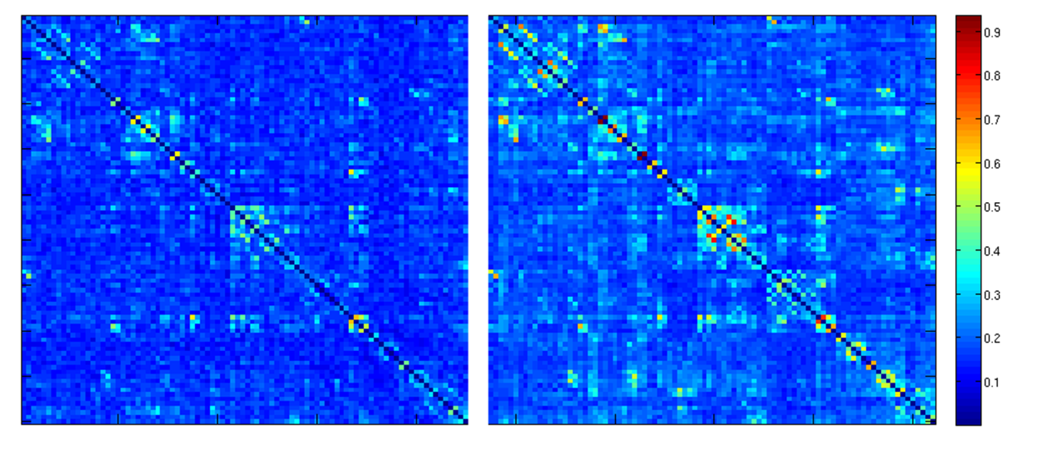

Supplement: Figure S1 — Mean z-score matrices for normal and AD group. Each figure shows a 90×90 square matrix, where the x and y axes correspond to the regions listed in Table S1, and where each entry indicates the mean strength of the functional connectivity between each pair of brain regions. The diagonal running from the upper left to the lower right is intentionally set to zero. The z-score of the functional connectivity is indicated with a colored bar. (TIF) [file pone.0033540.s001.tif]

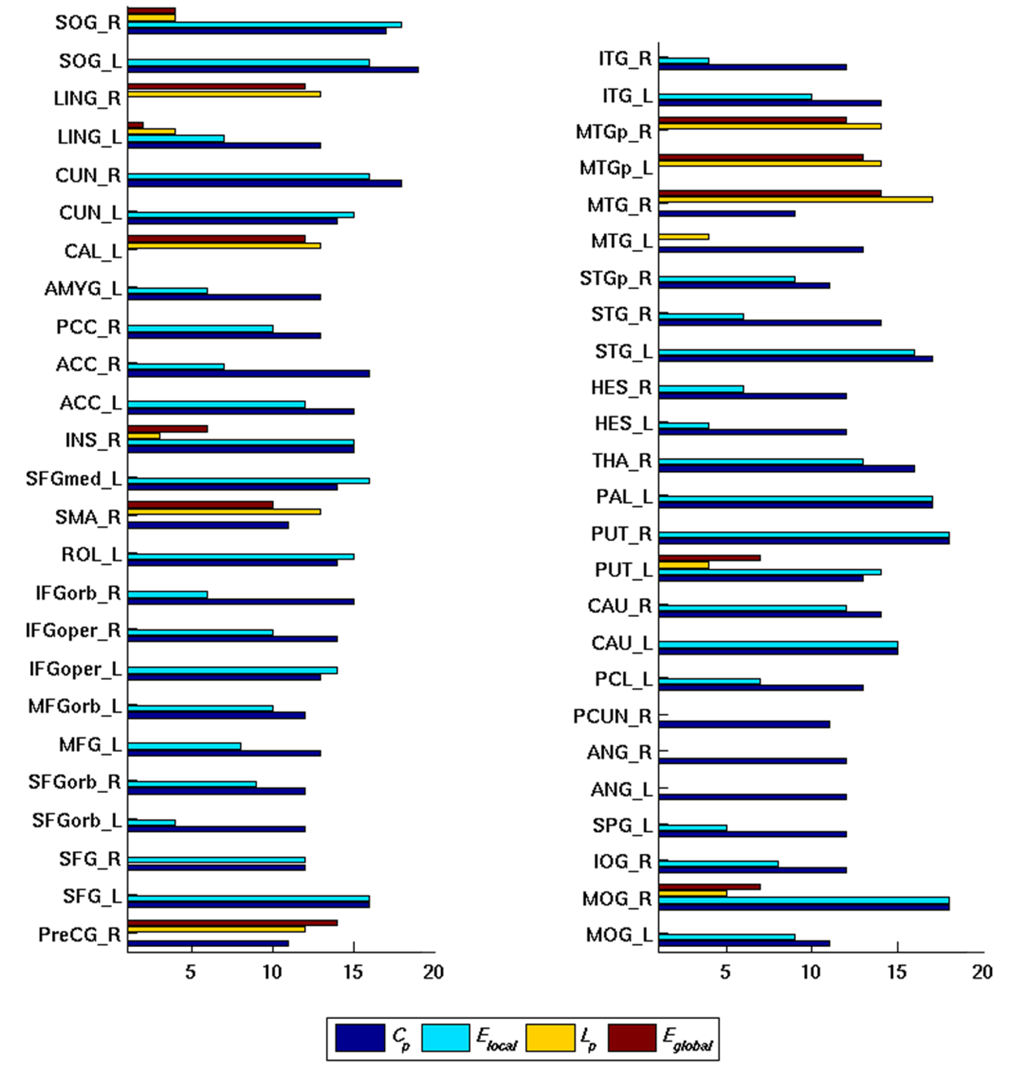

Supplement: Figure S2 — The frequencies distributions of altered brain areas in AD group of the 19 different thresholds. Y axes presents significantly altered brain areas. The traverse axes correspond to the frequency of significant differences happened between the two groups from 0.04–0.40 at 0.02 intervals. Clustering coefficient (blue), local efficiency (green), shortest path length (yellow), and global efficiency(brown). (TIF) [file pone.0033540.s002.tif]

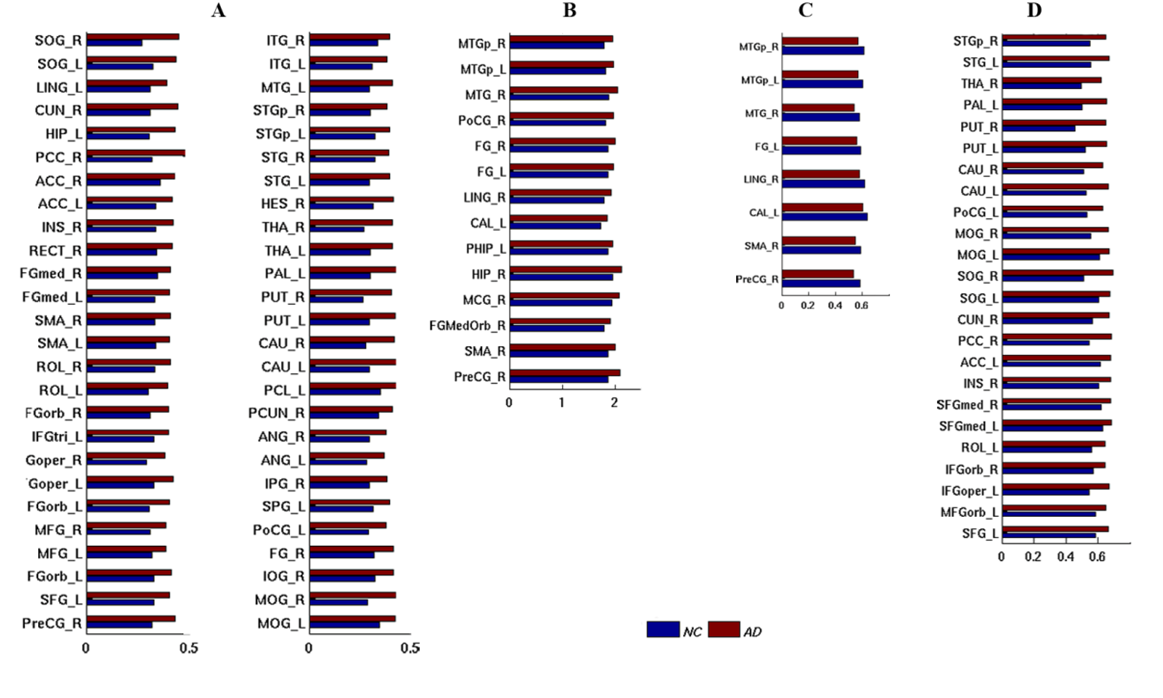

Supplement: Figure S3 — The altered brain areas between AD group (red) and NC group(blue) at the cost of 22%. A, clustering coefficient; B, shortest path length; C, global efficiency; D, local efficiency. (TIF) [file pone.0033540.s003.tif]

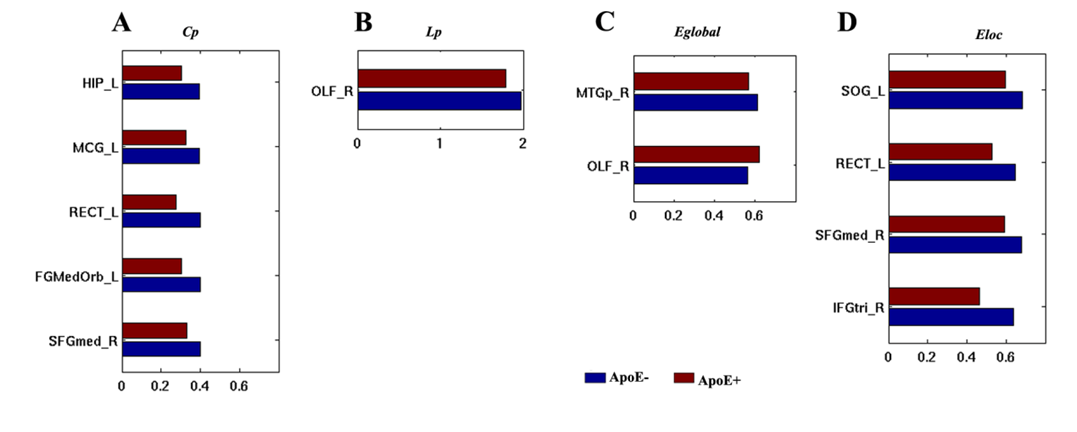

Supplement: Figure S4 — Brain areas showed significant alteration in network properties between ApoE+(red) and ApoE−(blue) groups in AD at the cost of 22%. A, clustering coefficient; B, shortest path length; C, global efficiency; D, local efficiency. (TIF) [file pone.0033540.s004.tif]
