# Supplementary material for: Disrupted Small-World Brain Networks in Moderate Alzheimer's Disease: A Resting-State fMRI Study
Source: PLoS One. 2012 Mar 23;7(3):e33540. doi: 10.1371/journal.pone.0033540 (PMC3311642; doi:10.1371/journal.pone.0033540)
Supplement: Table S1 — Cortical and subcortical regions defined in Automated Anatomical Labeling template in standard stereotaxic space. (DOCX) [file pone.0033540.s005.docx]

| **Region Name** | **Abbreviation** | **Region Name** | **Abbreviation** |
| --- | --- | --- | --- |
| Precentral | PreCG | Lingual | LING |
| Frontal_Sup | SFG | Occipital_Sup | SOG |
| Frontal_Sup_Orb | SFGorb | Occipital_Mid | MOG |
| Frontal_Mid | MFG | Occipital_Inf | IOG |
| Frontal_Mid_Orb | MFGorb | Fusiform | FG |
| Frontal_Inf_Oper | IFGoper | Postcentral | PoCG |
| Frontal_Inf_Tri | IFGtri | Parietal_Sup | SPG |
| Frontal_Inf_Orb | IFGorb | Parietal_Inf | IPG |
| Rolandic_Oper | ROL | SupraMarginal | SMG |
| Supp_Motor_Area | SMA | Angular | ANG |
| Olfactory | OLF | Precuneus | PCUN |
| Frontal_Sup_Medial | SFGmed | Paracentral_Lobule | PCL |
| Frontal_Mid_Orb | FGMedOrb | Caudate | CAU |
| Rectus | RECT | Putamen | PUT |
| Insula | INS | Pallidum | PAL |
| Cingulum_Ant | ACC | Thalamus | THA |
| Cingulum_Mid | MCG | Heschl | HES |
| Cingulum_Post | PCC | Temporal_Sup | STG |
| Hippocampus | HIP | Temporal_Pole_Sup | STGp |
| ParaHippocampal | PHIP | Temporal_Mid | MTG |
| Amygdala | AMYG | Temporal_Pole_Mid | MTGp |
| Calcarine | CAL | Temporal_Inf | ITG |
| Cuneus | CUN |  |  |

# Table S1.

Cortical and subcortical regions defined in Automated Anatomical Labeling template in standard stereotaxic space

Note: The abbreviations listed are those used in this paper, which differ slightly from the original abbreviations by Tzourio-Mazoyer et al. [[1](#_ENREF_1)]
